# Selections and Abstracts

**Published:** 1915-06

**Authors:** 


### SELECTIONS AND ABSTRACTS

### EARLY DIAGNOSIS OF CANCER OF THE UTERUS; OPERATIVE TECHNIC.

By Thomas S. Cullen, M. B., Baltimore, Md.

In the various portions of the mucous membrane of the uterus three definite varieties are to be found. The mucosa, covering the vaginal portion of the cervix, is made up of squamous epithelium; that between the external and internal os consists mainly of racemose or branching glands which secrete a varying quantity of mucus; whereas in the portion that lines the cavity of the uterus are numerous tubular glands differing totally from those of the cervix. From each of these types of mucosa cancer may develop.

In cancer of the uterus there occurs an outgrowth from the surface of the mucosa, while at the same time the growth penetrates into the underlying tissue. The cancerous tissue in the beginning is so rich in blood vessels that the slightest disturbance of the growth is liable to cause bleeding, and not infrequently an increased blood pressure is sufficient to bring about a faint show. As the growth advances, the older and more friable portions become necrotic and there results a breaking down which gives rise to a watery discharge, often tinged with blood and frequently fetid. This discharge is usually the first symptom of cancer, but it may be totally wanting until the growth has reached large proportions. Cancer of the uterus is most common between the thirty-fifth and fiftieth years, but it is occasionally noted in patients under twenty-five years of age.

Any bloody or watery vaginal discharge that can not be definitely accounted for demands an immediate and careful local examination. If on bimanual examination the cervix is found to be rough, friable and bleeding, the diagnosis of cancer is usually certain; but if the cervix is still intact, the diagnosis may be very difficult. In early carcinoma of the cervix, when no disintegration has occurred, the surface is usually nodular, and springing from it are fine finger-like outgrowths which bleed readily.

In every case a careful history should be taken but, even after all possible data have been obtained and after a thorough bimanual examination, it will not rarely happen that the physician can not determine to his satisfaction whether malignancy exists or not. In such cases a wedge of the suspicious area (about one cm. deep and two or three mm. broad) should be cut out, dropped at once into a ten per cent formalin solution and sent to the pathologist, who in the course of a few days will be able to decide with almost absolute certainty whether cancer is present or not.

When the cervix, on bimanual examination, appears normal, the lesion is usually situated in the cervical canal or in the cavity of the uterus. The finding of an enlarged and nodular uterus renders it probable that myomata are present. When myomata are of the submucous variety, the monthly periods are usually excessive, but as a rule no intermenstrual bleeding occurs and no fetid discharge exists, unless sloughing of a submucous nodule is going on. In the latter case, however, portions of the growth are apt to be found projecting from the cervix, and such sloughing masses are readily distinguishable from cancer of the cervix, because they are tough and not friable, and because the finger can be swept around them, proving that they have originated at a point higher up.

Extrauterine pregnancy frequently gives rise to intermenstrual bleeding and occasionally to a slight menstrual discharge, but in these cases we usually have a history of a missed period or of a period that has persisted, and in addition there is gradual distention of the tube. Finally, the bimanual examination will often reveal the definite, velvety mass to one side of the uterus.

Pelvic inflammatory conditions are at times accompanied by a bloody or watery vaginal discharge. In these cases we can usually learn that there has been some recent local vaginal infection or that an old pelvic lesions has recently been rekindled. In such cases there is often more or less elevation of temperature, whereas in early cancer there is no fever.

When the patient is stout, a satisfactory bimanual examination is often impossible, unless an anesthetic is employed. When the cervix feels normal and we have excluded myomata of the uterus and lesions of the adnexa, the cause of the bleeding usually lies in the cervical canal or in the cavity of the uterus. Whatever the uterine growth, it must drain into the uterine canal, otherwise there would be no vaginal discharge.

In such cases the uterus should be most thoroughly cureted. The mucosa should be brought away from the anterior, posterior and lateral walls and likewise from the cervical canal. If much tissue is obtained, it is probable that malignancy exists. All of this tissue, including the blood, should be thrown into a ten per cent formalin solution without preliminary washing and sent to the pathologist.

In some cases the pathologist has considerable difficulty in saying whether a given specimen is cancerous or not, but as a rule, there is just as much difference under the microscope between cancerous and healthy mucosa as there is between two totally different patterns of wall paper. Although there is always a possibility of error, so exact is the aid obtained from the microscope in the examination of scrapings that in every instance during the last sixteen years in which we have made a microscopic diagnosis of cancer and have later examined the uterus, definite microscopic evidence of cancer was present. Naturally, a thorough knowledge of the various pictures due to faulty hardening of the tissues, to gland hypertrophy with or without pregnancy, and those peculiar to the normal mucosa, in early life, during menstruation and in old age, is necessary before one can undertake to pass judgment on the character of scrapings.

From no other part of the body is it possible to so easily obtain material for diagnosis. Take, for instance, cancer of the stomach; how thankful the operator would be were it possible to just introduce a straight curet to the pylorus and bring away some tissue for diagnosis, without the necessity of making any incision or of doing any suturing. For the early diagnosis of cancer of the stomach, an exploratory operation is usually necessary. We as general practitioners and surgeons have absolutely no excuse for failing to diagnose cancer of the uterus within one week after the first time the patient comes under our observation.

THE BEST OPERATIVE TECHNIC.

To speak of the operative technic for cancer of the uterus before a Pennsylvania audience is akin to bringing coals to Newcastle when among others of your number an old friend of mine, Dr. John Clark, has contributed so much to our knowledge of the subject. The operation as elaborated by Wertheim seems to offer the best results. It consists in the removal of the uterus, appendages and parametrium and often also of the pelvic lymph glands.

It is now generally recognized that the greatest dangers of the radical operation are due to the shock that immediately follows. More recent experience has shown that it is possible to lessen this shock in two ways, (1) by shortening the duration of the operation, (2) by keeping the patient warm while she is on the table and afterwards.

With the abundant flood of "sunshine," as furnished by Kronig's light, the operator can often save from fifteen to thirty minutes. This light should be in every operating room where much abdominal work is done.

The chief bleeding encountered in the radical operation is from the vaginal plexus of veins. The long, short-curved Wertheim forceps enables one to clamp and cut the vessels with great facility, thereby often saving from ten to fifteen minutes. As was said before, this shortening of the operation by even ten minutes is all important.

By means of Kronig's electrically heated table the patient can be kept at an equable temperature and usually leaves the operating room in an infinitely better condition than when the ordinary table is used. The patient is cleaned up on the regular table, and after being thoroughly dried is placed on double blankets laid over the electric table. It is always necessary to have a special nurse or assistant guard against any danger of burning the patient, and the current should be turned on and off as necessary in order that a reasonably equable temperature may be maintained. In the course of a few weeks I operated on six cancer patients in succession and, although in some of them the operation was a most extensive one and the patients were weak, in not one case did there occur the marked degree of post-operative shock so often noted.

In the time at my disposal I have merely sketched the salient features in the diagnosis and treatment of cancer of the uterus and have omitted any consideration of polypi, adenomyoma, sarcoma and chorio-epithelioma.

In conclusion, I can not refrain from quoting an appeal made by the late J. Knowsley Thornton some years ago, at once an appeal to us as medical men and one of the severest arraignments of our profession that has ever been made in our management of cancer of the uterus:

How is an early diagnosis to be made? Clearly by neglecting no menstrual departure from the normal, however trivial it may at first sight appear, but at once to encourage the patient to accurately describe symptoms, and above all to insist in the most determined manner on a local examination. Here it will be apparent that I, as a consultant, appeal for help to the great body of those who are now listening to my remarks, to my professional brethren engaged in general practice. I, in common with those situated as I am, too seldom have an opportunity of diagnosing early, because the majority of the patients come to us too late, when the disease has already advanced nearly or quite beyond the limits of surgical aid. Let me then appeal to all engaged in family practice who listen to me here, and to that larger body who may read my words when reproduced in the medical journals, to sternly cast aside that too great modesty or that tendency to treat as trivial small symptoms, and to at once take alarm about, and carefully investigate, every case in which there is brought to their notice an abnormality in menstruation, or a vaginal discharge of any kind, however trifling. A very grave responsibility lies at the doors of the medical profession for the small progress made in the early diagnosis of uterine cancer and its successful treatment. How constantly is the consultant told: "I mentioned it to my doctor weeks or months ago, but he said, 'Oh, it is nothing; I will send you a little medicine or a little injection' and never even suggested any internal examination, so I did not like to trouble him again till the pain became so bad or the discharge so troublesome, and then he examined me and said I must have special advice at once"? Invaluable weeks or months gone, and then the verdict of the consultant: "It is not a case for operation," which really means "You have come too late," but can not be so candidly expressed, because he must guard against the reputation of his professional brother. I admit that the false modesty of the patient, especially in some classes of society, makes the position a difficult one, especially for the young family doctor, but let me implore you all to awake to what is at stake, and to be firm in your demand for an examination, and if you have any doubt after such an examination, to urge that the patient should at once seek the advice of some one who has larger opportunities than yourself for forming a sound opinion. I will go one step further, and ask you, if there should be any to whom such a temptation comes, never to go on treating a case in which there is a shadow of doubt, either because you doubt or because you want practice; if the case is susceptible of treatment at all, it is only surgical treatment which can avail, and that of so severe a kind that it requires the knowledge of the specialist if ever any disease did or does require special knowledge and special skill in operative treatment.

---

### EDUCATION OF THE PUBLIC CONCERNING CANCER OF THE UTERUS.

By John G. Clark, M. D., Philadelphia.

In addressing the public on questions of health, data bearing upon those diseases in which great progress has been made may be used with telling force. Thus, the remarkable improvements in the prevention and cure of variola, typhoid fever, malaria, yellow fever, diphtheria, etc., leave little of an enigma; for the etiology and pathology are now perfectly understood.

With a disease like cancer, however, we have not this incisive data upon which to base public instruction. The most the optimist can offer, notwithstanding the immense volumes of fruitless research, would be summarized in one short sentence: The earliest possible diagnosis and the most radical removal. The pessimist might even smile at this sentence and attempt to maintain the statement of the great surgeon, Sir James Paget, who many years ago summarized his observations as to permanent cure as follows: "I will not say that such a thing as cure is impossible, but it is so highly improbable that the hope of this occurring in a single instance can not be reasonably entertained."

Child, who has recently reviewed this subject in the British Medical Journal, after quoting Sir James' fateful sentence, carries us forward to the present and, while not enthusiastic, indicates progress. He says, "While melancholy enough as is the picture of our treating cancer at the present time, it must be admitted that it forms a forcible contrast with that just portrayed." The most authentic statistics are now irrefutable as to cures in cancer, not in isolated instances but in figures ranging in various organs from ten to fifty per cent. We have, therefore, made very decided progress, but, measured by our hopes for the future, the results are indeed meagerly pathetic and show that for every victim rescued thousands pass to death through more enduring and excruciating tortures than those suffered by the most wretched victims of the Blood Council. We are happily in a transitional period, and the optimist may cheerfully work with the tools now in hand, confidenly hoping that out of the seething caldron of research sooner or later the radical cure will come.

### EDUCATE FIRST THE PHYSICIANS.

Before turning to the laymen of this educational question let us be sure that members of our own guild are blameless.

How often is the lump in the breast treated expectantly, and with what tragic frequency are women assured, even in the presence of the most evident signs of menstrual disturbances, that the ailment is insignificant, or at most of a transitory character. It is wise, therefore, for us to look to our own professional skirts before assuming the function of public laundress.

Publicity will, however, work two ways: First, in instructing women as to the significance of untoward menstrual symptoms, particularly at the climacteric epoch; and, second, the indolent in our profession will be so shocked by the roorback from this propaganda that he will give closer heed to symptoms which the layman has learned to regard with suspicion. This has been noted already in Germany, where the instruction of the public has been instituted through popular articles in lay periodicals. Impressed by the overwhelming number of patients with inoperable carcinomata seeking care at the German clinics, Winter in 1891 instituted an investigation to determine the cause and whether it was remediable. He found on careful inquiry that thirty-three per cent had been treated for considerable periods of time without examination, the physicians simply relying upon a very cursory history for his guide in treatment. As a result of this appalling observation a vigorous campaign against such haphazard and criminal methods was instituted, which quickly wrought a gratifying improvement. Four years later, in the same locality in Eastern Prussia, he reviewed one hundred clinic cases with the following results: Eighty-seven patients had been examined at once; nine had been referred to a clinic for examination; and ten only had been treated without an examination. Thus there had been a decrease of from thirty-three to ten per cent in four years. A quick response to a worthy impulse.

Coincident with this improvement a very decided increase in operability was noted. Up to 1891 only thirty-two per cent were within the surgical pale; in 1895, this ratio had increased to forty-two per cent. While this salutary influence was yielding its return among the medical profession, there was noted even a happier result among the women who were being reached by popular instruction.

